# P-1415. Infectious Causes of Classical Fever of Unknown Origin: A Prospective Observational Study from a Tertiary Care Centre in North India

**DOI:** 10.1093/ofid/ofaf695.1602

**Published:** 2026-01-11

**Authors:** Md Tariq Maula, Sandeep Rao Kordcal, Piyush Ranjan, Ashutosh Biswas, Naveet Wig

**Affiliations:** All India Institute of Medical Sciences, New Delhi, Delhi, India; All India Institute of Medical Sciences, New Delhi, Delhi, India; All India Institute of Medical Sciences, New Delhi, New Delhi, Delhi, India; All India Institute of Medical Sciences, New Delhi, Delhi, India; All India Institute of Medical Sciences, New Delhi, Delhi, India

## Abstract

**Background:**

Fever of unknown origin (FUO) is difficult to diagnose despite recent improvements, because of its nonspecific clinical characteristics and wide differential diagnosis. Although infections are the most common causes in underdeveloped nations, they are frequently overlooked in their early stages because of atypical presentations and empirical use of antibiotics. The purpose of this study was to describe the clinical characteristics, diagnostic methods, and infectious spectrum of patients with classical FUO.

**Figure 1:**
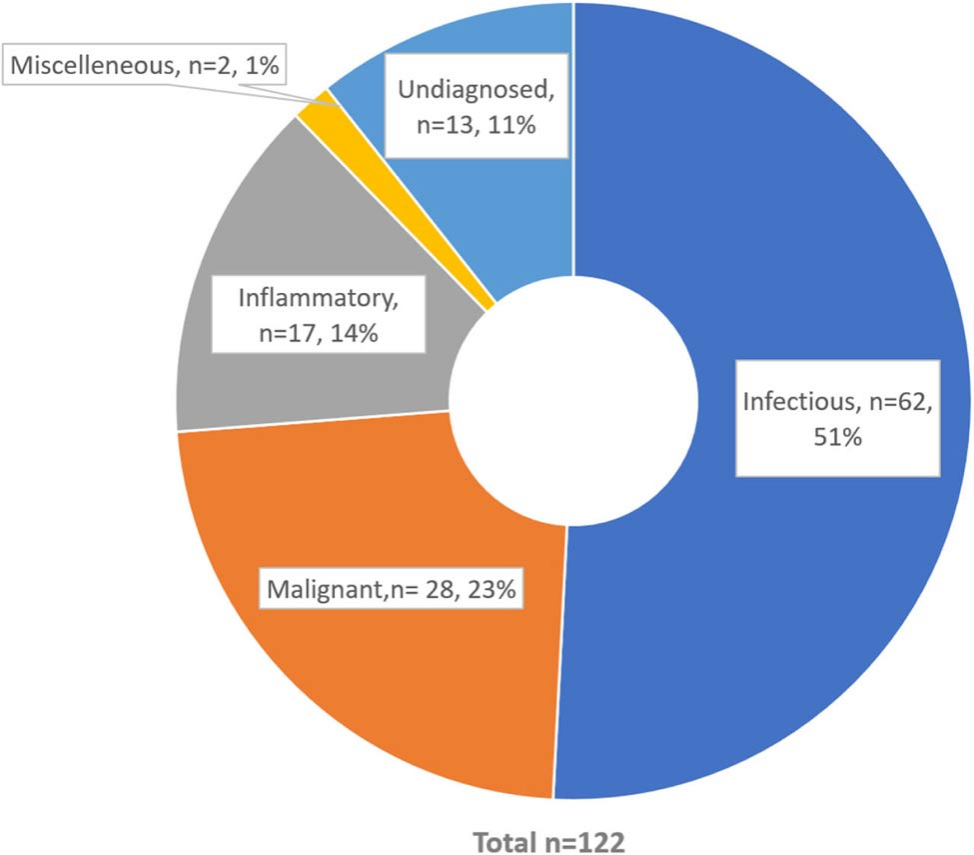
Etiological distribution of FUO cases

**Figure 2:**
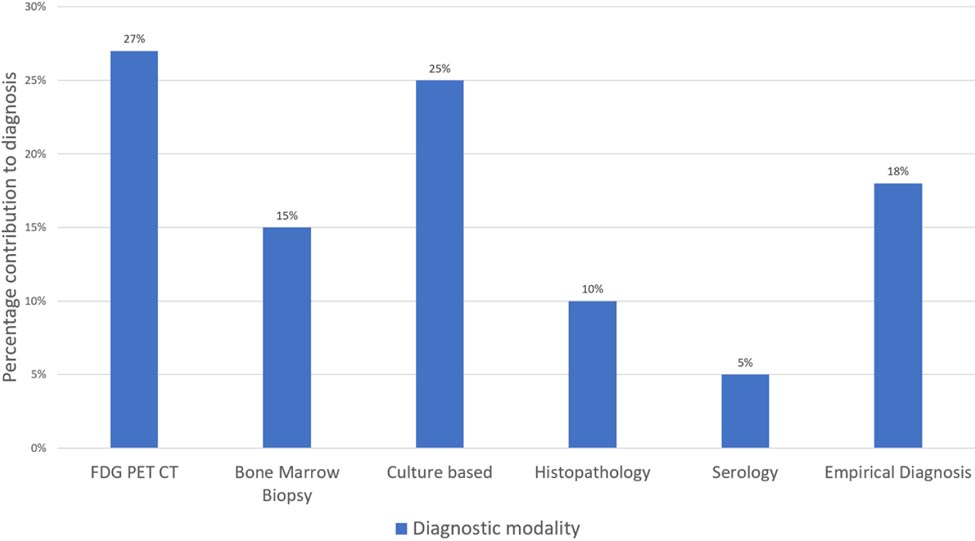
Diagnostic yield of investigations in infectious FUO

**Methods:**

We prospectively enrolled 122 adult patients meeting classical FUO criteria at a tertiary care center in North India. Comprehensive data, including clinical, laboratory, imaging, course, and clinical outcome, were collected. Final diagnoses were categorized as infectious, inflammatory, malignant, miscellaneous, or undiagnosed. Subgroup analysis was done focusing on infectious etiologies.

**Table 1:**
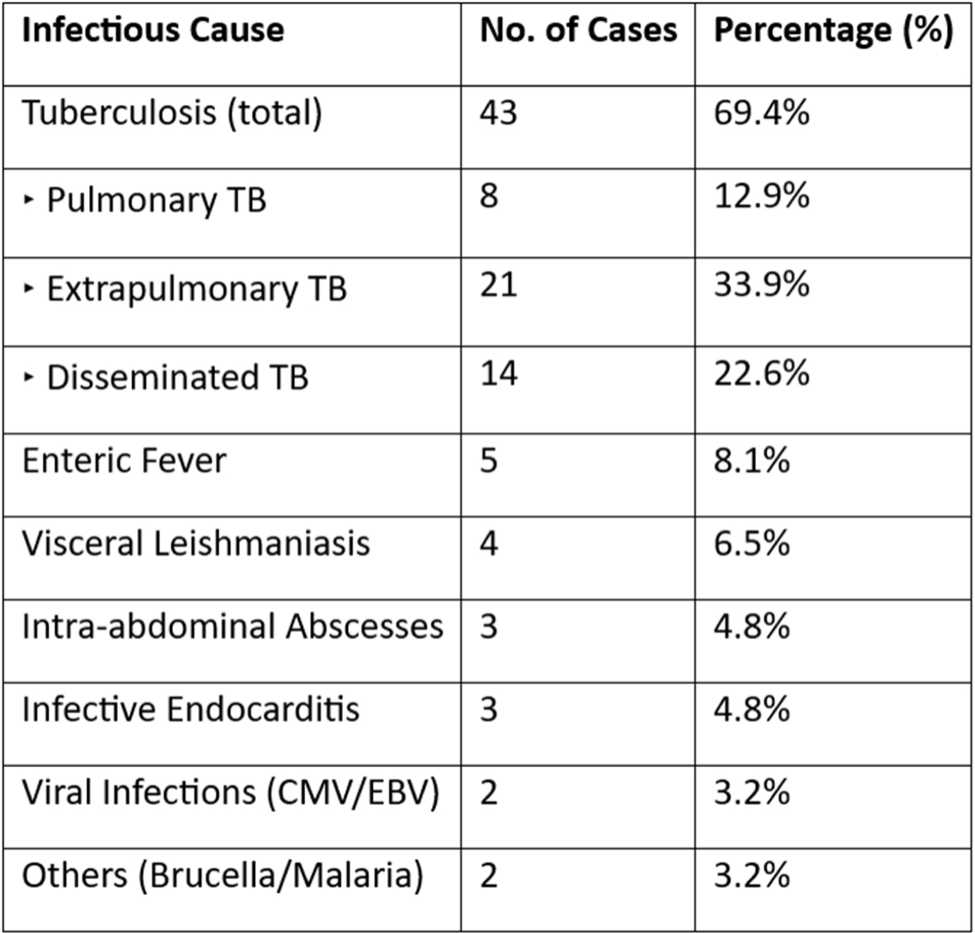
Spectrum of infectious etiologies in FUO

**Results:**

Infections accounted for 62 of 122 cases (50.8%), while other causes included malignancy(n=28, 23%), inflammatory(n=17, 13.9%), miscellaneous (n=2, 1.6%), and 13(10.7%) cases remained undiagnosed. The most common infectious cause was tuberculosis(n=43/62, 69.4%), which included pulmonary (12.9%), extrapulmonary (33.9%), and disseminated forms (22.6%). Additional infectious causes included enteric fever (8.1%), visceral leishmaniasis (6.5%), intra-abdominal abscesses (4.8%), infective endocarditis (4.8%), and viral infections (CMV/EBV, 3.2%). Among patients with infection, the median length of fever was two months. In 42% of these instances, a bone marrow sample plus an FDG PET-CT scan helped with the diagnosis. Infectious FUO had a considerably higher prevalence of prior antibiotic usage (p=0.037), which may have decreased microbiological output. The percentage of mortality among infectious cases was low(1.6%).

**Conclusion:**

Infectious diseases, particularly tuberculosis, are still the leading cause of classical FUO in endemic settings. Early consideration of extrapulmonary and disseminated TB is vital. Advanced diagnostics such as PET-CT and bone marrow examination substantially improve etiological yield. Judicious antibiotic use is crucial to avoid masking microbiological diagnoses and ensuring timely, targeted therapy.

**Disclosures:**

All Authors: No reported disclosures

